# Psychometric properties of Bengali version of QOLIE-10 in epileptic patients

**DOI:** 10.4103/0972-2327.40222

**Published:** 2008

**Authors:** Sagar Basu, Debasish Sanyal, Malay Ghosal, Biman Roy, A. K. Senapati, S. K. Das

**Affiliations:** Bangur Institute of Neurosciences and Psychiatry, Kolkata - 700 025, India

**Keywords:** Epilepsy, QOLIE-10, SRQ 24

## Abstract

Assessment of Quality of Life in Epilepsy has currently been emphasized to provide comprehensive care to patients.

**Aim:**

To develop and standardize and assess the psychometric properties of Bengali version of QOLIE-10 and to assess the relationship of quality of life with seizure variables and presence of psychiatric morbidity.

**Design:**

English QOLIE-10 was translated into Bengali by a translation committee using translation-re-translation technique. Inter-rater reliability between the English and Bengali version was assessed during initial practice session held amongst 20 bilingual patients. It was found that item 3 (related to driving) was reported to have difficulty in answering by all the patients as none drove any vehicle. Thus, this item was dropped. The inter-rater reliability of the resultant 9 item scale was found to be high (kappa = 0.9). One hundred and seven epilepsy patients attending the Epilepsy clinic were selected for the study if they met the following criteria: age >15 years, duration of seizure >1 year, regular intake of antiepileptic drugs, presence of informant and ability to read Bengali. For each patient, demographic and clinical data (seizure frequency, last seizure date, seizure type as per record, medicine intake history and records of past investigations such as EEG) was collected. Each patient were administered QOLIE-9 (Bengali) and SRQ-24 Bengali version to screen for psychiatric morbidity.

**Results:**

The Cronbach's Alpha coefficient for QOLIE-9 was 0.81, which did not improve if any item was dropped. All items showed strong correlation with the total score. The instrument showed stable factor structure with three factors (Limitation, Depression, Illness effects). However, the item with regard to memory problem did not fit into any of the factors. The QOLIE-9 total showed a significant correlation with the seizure frequency (r = 0.76**). SRQ positive (i.e., suspected psychiatric morbidity) cases had higher QOLIE-9 score (thus, poorer quality of life) in comparison to non-psychiatric cases.

**Conclusion:**

Bengali QOLIE-9 is a valid and reliable instrument to assess the quality of life in patients suffering from epilepsy.

In 1990, the analysis conducted by World Health Organization on worldwide burden of diseases, epilepsy ranked among the top three causes of neurological disability in developed countries, particularly among the young.[1] Epilepsy has an estimated age-adjusted annual incidence of 30 to 60/100,000 and a prevalence of 6/1,000.[2] Epilepsy affects 40 million people worldwide, three-quarters of who remain untreated. Approximately 75% of the 40 million people with epilepsy all over the world are in developing countries. Since the prevalence rate of active epilepsy in India is 5.5/1000, the number of active epilepsy patients in India will amount to 5.4 million, i.e., one-eighth of the total epilepsy patients in the world.[3] In spite of the huge magnitude of the problem, epilepsy can be controlled in three-quarters of the patients if early diagnosis and treatment is done.[4]

As epilepsy results in significant psychological and social consequences for everyday living,[5] the illness tends to have lifetime effects on the patient and the family. Jacoby[6] described epilepsy as “both a medical diagnosis and a social label.” The effects of epilepsy on the patient and the family depends on several factors, including the type and frequency of the seizures, the medication prescribed and its effects on the behavior and development of the individual and the social impact on the patient and family.[7] Dodrill *et al.*[8] suggested that seizures may be only one of the several variables that impact the psychosocial functioning of patients with epilepsy. They found that that vocational adjustment is the most frequently reported factor that is related to patient psychosocial outcome.

The measurement of the outcome of epilepsy treatment has traditionally assessed the seizure frequency and severity, adverse effects and antiepileptic drug levels. The perceptions of the patients often include additional parameters that encompass the effects of epilepsy on daily activities and functions.[9]

The quality of life (QOL) evaluation is a relatively new measure to evaluate the outcome of epilepsy. The QOL is influenced by biological factors as well as cultural, social and religious beliefs and values.[10] Many factors influence the QOL of people with epilepsy, including seizure severity, stigma,[11] fear and the presence of cognitive or psychiatric problems.

There has been considerable interest and concern regarding the effects of epilepsy on quality of life.[12] While most of such studies have been conducted in developed countries, studies in India[13] show the disruptive impact of epilepsy on daily lives of those with the condition.

The health-related QOL instruments for a population with epilepsy were developed from the questionnaires that were used for evaluating the general population. These instruments include the QOLIE-89 instrument, QOLIE-31 and QOLIE-10.[9,14]

The QOLIE-10[14] is a self-administered questionnaire, designed for completion by patients alone. It comprises of seven components: seizure worry, overall QOL, emotional well-being, energy-fatigue, cognitive functioning, medication effects (physical effects and mental effects) and social function (work, driving and social function). The scale items are scored in Likert style (1-5, low score implies better quality of life).

Studies suggested that shorter version such as QOLIE-10 was as effective as its longer counterparts.[9,14] Unfortunately, no local vernacular version was available to use QOLIE in our setup.

## Aims and objectives

1. To prepare a Bengali version of the QOLIE-10.
2. To establish the reliability and validity of the Bengali version of the QOLIE.
3. To explore relationship between QOLIE score and epilepsy related clinical features (seizure type, seizure frequency, occurrence of last seizure).

## Materials and Methods

All patients with epilepsy attending Epilepsy clinic were reviewed for inclusion in the study.

Inclusion criteria were as follows:

1. Age >15 years
2. Duration ≥1 year
3. Regular records of seizure (seizure diary)
4. Regular intake of antiepileptic drugs.
5. Ability to understand Bengali.
6. Willing to participate in the study
7. Presence of informant (spouse/parent/near-relative).

Exclusion criteria were as follows:

1. Presence of any serious medical/surgical problem not related with epilepsy.
2. Mental retardation/overt cognitive impairment.
3. Refusal to participate in the study
4. Irregular follow-up, poor seizure record keeping, non-compliance with treatment.
5. No informant available.

### The translation of QOLIE-10

QOLIE was translated in Bengali by the authors and independently by two bilingual teachers of Bengali. These translations were pooled together, and a final version was prepared. The actual meaning of the question was given more emphasis in comparison to the word-to-word translation. This Bengali version was then retranslated into English by two bilingual health professionals who had not seen the original English version. The translation-retranslation reliability was found to be satisfactory when two English versions were compared. The final Bengali version was arrived at by a consensus decision by all the translators with attention being paid to content, semantic, technical and conceptual equivalence of the Bengali version. Practice session was held on 20 patients to assess ease of understanding by patients. Item number 3 (“has your epilepsy or antiepileptic medications caused trouble with driving?”) was reported to be difficult to be answered by all the patients as none of them drove any vehicles. Thus, this item had to be dropped. Then, the agreed upon questionnaire (QOLIE-9 Bengali) and the English questionnaire (item 3 omitted) was administered to a bilingual cohort of approximately 20. Half the cohort received the Bengali version first and half the English. Later that day or the next day, other version was provided to each subject. The inter-rater reliability was assessed during this process.

The inter-rater reliability was found to be high (kappa for all the items was above 0.9). No further difficulties were found in the case of any of the items. This Bengali version was then used for the present study.

For each patient, demographic and clinical data (seizure frequency, last seizure date, seizure type as per record, medicine intake history and records of past investigations such EEG) was collected. Each patients were administered QOLIE along with SRQ-24.[15]

The results were tabulated and analyzed using statistical program SPSS ver9. The demographic data was summarized using descriptive measures. Scale data was reported as an item and entire scale summary, internal consistency measure (Cronbach's Alpha), item-item and item total correlation measure. The presence of any hidden construct in the scale was assessed using Principal Component Analysis (PCA). In order to determine the minimal loading required for an item to be a part of a factor, we used[16] alternative formula for minimal loadings when the sample size, N, is 100 or more: Min FL = 5.152/ [SQRT(N-2)]. Here, n = 107 and minimum loading = 0.5.

## Results

The study involved 107 subjects (Mean age: 26.24 ± 8.45 years, Median age: 25 years) with a slightly higher number of female patients (57) in comparison to males (50). Female subjects were slightly older (26.3 ± 8.6 years, median age: 25 years) than males (mean age: 26.18 ± 8.4 years, median age: 24 years), although the difference was not statistically significant (t = 0.072, d.f = 105, *p* = 0.943, N.S.). Most patients were unmarried (63.6%) with the proportion of unmarried patients being higher in females (66.7 %) when compared to that in males (60%). Most patients (58.9%) came from urban areas. Majority of patients (51, i.e., 47.7%) were educated up to middle school level.

### Information from Table 3A and 3B of First draft paper now incorporated in the paper text

**Table 3:** Distribution of QOLIE-9 (Bengali version) total score according to presence/absence of psychiatric morbidity

	Number of subjects	QOLIE-9 (Bengali version) total score	
		
		Mean	Standard deviation
Psychiatric morbidity absent	36	17.17	3.78
Psychiatric morbidity present	71	23.58	6.21
Total	107	21.42	6.28

Mann-Whitney U = 580.5, *p* = 4.1 × 10^−6^: 71, i.e., 66.4% patents had psychiatric morbidity

As expected in a tertiary care facility, most patients had high rates of seizure (Mean number of seizures: 12.8 ± 6.8, median number of seizures: 12). A large number of patients had seizures on the day of assessment (thus, the last seizure occurred zero days back), the median duration being 15 days.

### QOLIE-9 total score showed a significant correlation with seizure frequency (r = 0.76**)

The internal consistency for the QOLIE-9 overall scores was high. The Cronbach's Alpha coefficient for QOLIE-9 was 0.81, which did not increase if any item was dropped.

The PCA with varimax rotation was performed. The Min FL = 5.152/[SQRT(N - 2)] and minimum loading = 0.5

Table 1 shows the correlation of individual items with QOLIE-9 total. Clearly, all the items show strongly significant correlation with QOLIE-9 total, thereby indicating that none of the items are apparently misfit in the scale.

**Table 1:** Pearson correlation coefficients for the components of QOLIE-9 (Bengali version) (*n* = 107)

QOLIE9 item	Pearson correlation coefficient with QOLIE9 total score	Two-tailed significance of pearson correlation coefficient
Item 1 - Energy level	0.631	*p* < 0.001***
Item 2 - Felt blue	0.466	*p* < 0.001***
Item 3 - Memory problem	0.573	*p* < 0.001***
Item 4 - Work limitation	0.702	*p* < 0.001***
Item 5 - Social limitation	0.634	*p* < 0.001***
Item 6 - Physical effect	0.575	*p* < 0.001***
Item 7 - Mental effect	0.476	*p* < 0.001***
Item 8 - Fearful of having fit	0.754	*p* < 0.001***
Item 9 - How things are going	0.846	*p* < 0.001***

Table 2A and B shows that QOLIE-9 has stable factor structure with 3 factors. By using the factor naming methods, as suggested by Pet *et al.*,[17] (e.g., using item with the highest loading on a factor as the clue for the possible name, the meaningfulness of the name in conveying the meaning/ congruence of items) and the three factors were as follows: I - Limitation, II - Depression, III - Illness effects. Interestingly, the item related to memory problems did not fall within any of these factors. However, as this item was fulfilling other psychometric features, we decided to keep it in the scale.

**Table 2A:** Factor structure of QOLIE-9 (Bengali version) - principal component analysis with varimax rotation

Component	Initial eigen values		Rotation sums of squared loading	
		
	Total	Percentage of variance	Total	Percentage of variance
1	3.720	41.337	2.87	31.887
2	1.394	15.487	1.75	19.446
3	1.097	12.191	1.591	17.682
4	0.862	9.58		
5	0.615	6.829		
6	0.462	5.136		
7	0.363	4.029		
8	0.265	2.95		
9	0.222	2.462		

**Table 2B:** Item composition of the three factors obtained from QOLIE-9 Bengali version using principal component analysis with varimax rotation

Items of QOLIE-9 Bengali version	Factor I	Factor II	Factor III
Item 1 - Energy level		0.78	
Item 2 - Felt blue		0.92	
Item 3 - Memory problem			
Item 4 - Work limitation	0.825		
Item 5 - Social limitation	0.856		
Item 6 - Physical effect			0.694
Item 7 - Mental effect			0.866
Item 8 - Fearful of having fit	0.692		
Item 9 - How things are going	0.799		

All items of the scale (except Item 3 - Memory problem) load to any one of the three factors; no item is shared by two or more factors

Table 3 shows that SRQ positive (i.e., suspected psychiatric morbidity) subjects had higher QOLIE-9 total score (thus, poorer quality of life) as compared to nonpsychiatric cases

## Discussion

This study shows that the shorter versions of QOL instruments such as QOLIE-9 provides valid and useful information that supplements the general clinical data conventionally used to monitor epilepsy patients.

The Bengali translated version of QOLIE-9 was found to be valid and reliable in the local context. A strong relationship between various clinical parameters such as seizure frequency and presence of psychiatric morbidity indicate the clinical validity of QOLIE-9 and that instrument does serve as a comprehensive measure of the life of epilepsy patients. The finding of the present study is that the QOL falls with increased seizure frequency, as reported in the study of Thomas *et al.*[10] Other studies indicated that people with well-controlled seizures are less likely to report psychosocial problems and people in seizure remission (>2 years seizure-free) report a QOL that is not significantly different from those without the condition.[18] However, the specific three factors found in this study are unique and may reflect the unique way in which the patients of this sociocultural milieu perceive the QOL. As such, it is very important to explore local perception;[11] further studies are required to test these factors.

In the earlier studies, several factors have been identified to significantly correlate with QOL; seizure frequency,[19] presence of adverse effects,[18] female gender,[19] low educational status and psychosocial factors are some of them.[20]

The present study indicates that 71, i.e., 66.4% patents had psychiatric morbidity. Vuilleumier and Jallon[21] estimated that 20-30% of patients with epilepsy have psychiatric disturbances. Tucker[22] reported that one study found that 70% of patients with intractable complex partial seizures had one or more diagnoses consistent with *Diagnostic and Statistical Manual of Mental Disorders, Revised Third Edition (DSM-III-R)*-58% had a history of depressive episodes. Another perspective regarding the overall psychiatric morbidity seen in a modern comprehensive epilepsy center inpatients was reported by Blumer *et al.*[23] They found that 65% require psychiatric treatment - the largest proportion of patients with psychiatric disorder had a significant mood disorder (34%). In our study, we found that the QOL is lower in patients with psychiatric morbidity, which is not consistent with the findings of Lehrner *et al.*[24]

Hermann and Whitman[25] hypothesized three alternative groups of variables that contribute to the understanding of the impact of epilepsy: psychosocial variables such as fear of seizures, perceived stigma and discrimination; degree of adjustment to the diagnosis; other life events and level of social support; neurological variables such as age of onset, duration of epilepsy, seizure type and seizure severity; and medication variables, including medication type and number.

Shortcomings of the study:

1. The study comprised only 107 patients, which reduced the power of the overall study to detect the significant contributors that influenced the QOL. This problem also precluded the use of advanced statistical techniques such as regression to study the influence exerted by various seizure-related and demographic variables on QOL.
2. The effect of treatment of depression and/or better seizure management on QOL was not assessed.
3. Serum drug level estimation, which would have given more authentic picture of medicine intake (i.e., true compliance), could not be performed due to high cost.
4. As the patients have been recruited from a tertiary care centre, the selection of patients may have some bias as most of the patients belong to prognostically bad type.
5. The factor structure revealed during this study needs confirmation from larger studies and use of more advance statistical techniques such as Confirmatory Factor Analysis.

This study indicates that the QOLIE-9 can be used to screen patients in clinical settings and for clinical trials. Being simple questionnaires, it can be completed easily and quickly by patients. All the items in these questionnaires pertain to aspects of daily living for people with epilepsy; acceptance of this is likely to be high among patients and doctors. More frequent use in epileptic patients will help in a better understanding of the area of concern of the patient. The presence of high number of depression cases calls for better liaison between the neurologists and the psychiatrists. Increased attention to social and psychological consequences of epilepsy, as proposed by Sackellares and Berent,[26] will enable the provision of comprehensive care to such patients and enable the patients to live and cope with epilepsy better.
